# Speciation and Degrees of Contamination of Metals in Sediments from Upstream and Downstream Reaches along the Catchment of the Southern Bohai Sea, China

**DOI:** 10.3390/ijerph120707959

**Published:** 2015-07-14

**Authors:** Li Xu, Jing Li

**Affiliations:** 1 Beijing Research Center for Agricultural Standards and Testing, Beijing Academy Agriculture & Forestry Sciences, Beijing 100095, China; E-Mail: xuliforever@163.com; 2 Risk Assessment Lab for agro-products (Beijing), Ministry of Agriculture, Beijing 100095, China; 3 Key Laboratory of Ecosystem Network Observation and Modeling, Institute of Geographic Sciences and Natural Resources Research, Chinese Academy of Sciences, Beijing 100101, China

**Keywords:** Community Bureau of Reference (BCR), sediment quality guidelines, salinity, cluster analysis, correlation analysis

## Abstract

Environmental processes and biological community structures change along fluvial gradients within coastal river basins; the accumulation and associated risk of metal contamination would also be expected to change from upstream to downstream reaches. Speciation and degrees of contamination of metals in sediments from the upstream and downstream of river catchments of the southern Bohai Sea were investigated. The mean concentrations of Cu, Zn, Cr, Ni, Cd and Pb from upstream reaches were 82.6, 157, 63.6, 26.6, 0.18 and 24.9 mg/kg, respectively. The mean concentrations of Cu, Zn, Cr, Ni, Cd and Pb from downstream reaches were 38.0, 66.0, 38.9, 18.1, 0.16 and 24.0 mg/kg, respectively. Most of the Cu, Zn, Cr, Ni and Pb in sediments from both the upstream and downstream reaches was mainly associated with the residual fraction. However, Cd was preferentially bound to the exchangeable phase. A cluster analysis was used to study the degree of association between sites, and three distinct clusters were identified in both upstream and downstream sediments. A correlation analysis was conducted to determine the extent of association among metals and showed that metals in sediments from the upstream reaches have more affinity than those in the downstream area. Sediment quality guidelines were used to evaluate potential risks. The risks from Zn, Cr and Ni in the upstream reaches were higher than those from downstream reaches; however, the other three metals (Cu, Pb and Cd) showed opposite results.

## 1. Introduction

China is currently the world’s second largest economy as a result of rapid industrial development over the past few decades [1]. Rapid economic development has added huge loads of metal contaminants to rivers [2,3]. The watershed of the southern Bohai Sea is part of the Bohai economic rim and Yellow River economic belt, one of the main parts of China’s coastal economic belt [4]. Within the watershed of the southern Bohai Sea are about twenty rivers, numerous pollutant-generating plants, ten big cities and a population of tens of millions of people. Due to considerable urbanization and industrialization in the cities, the watershed of the southern Bohai Sea has been facing severe pollution problems, especially metal contamination. Because of industrial wastewater, domestic sewage and atmospheric deposition, a large amount of metals has been discharged into the rivers and then transported into the Bohai Sea. For example, the Yellow River, the second longest river in China, which has extremely high sediment loads, discharged 200 t of metals into the sea in 2003 and 778 t of metals in 2008 [5].

Sediments are the main repository and source of heavy metals in aquatic systems and play an important role in the transport and storage of potentially hazardous metals [6]. Trace metal contamination in sediments could affect water quality and the bioassimilation and bioaccumulation of metals in aquatic organisms, resulting in potential long-term implications for human health, as well as that of ecosystems [7]. However, the bioavailability and environmental behavior of metals in sediments are not only related to their total concentrations, but also are determined to a large extent by their chemical speciation [8]. Thus, the study of the speciation and distribution of metals in sediments has become one of the most important areas of environmental research [9].

Because environmental processes and biological community structures change along fluvial gradients within coastal river basins, the bioavailability and associated risk of metal contamination would also be expected to change from upstream to downstream reaches [10]. However, most previous studies have paid more attention to downstream reaches (namely marine and saline water systems). There is little information about the characteristic of metal contamination from the upstream reaches (namely freshwater systems), although the upstream pollution status plays an important role in determining the metal pollution downstream [11]. Furthermore, the chemical speciation of metals between upstream and downstream reaches within the catchments is largely unknown at the regional scale. The sequential extraction method [12,13,14] can provide information about the distribution of metal associated with different specific solid phases in sediments. The wide application of the sequential extraction protocols proposed by the Community Bureau of Reference (BCR) for metal speciation in river, marine, estuarine and stream sediments provided confidence for applying the BCR method in this study [15].

The objectives of this paper are: (1) to examine the characteristics of metal contamination in upstream and downstream reaches along the watersheds of the southern Bohai Sea, China; (2) to study the distribution of the speciation of metals in sediments from upstream and downstream reaches; and (3) to investigate environmental risks associated with metals from upstream and downstream reaches.

## 2. Materials and Methods

### 2.1. Sediment Collection and Preparation

**Figure 1:**
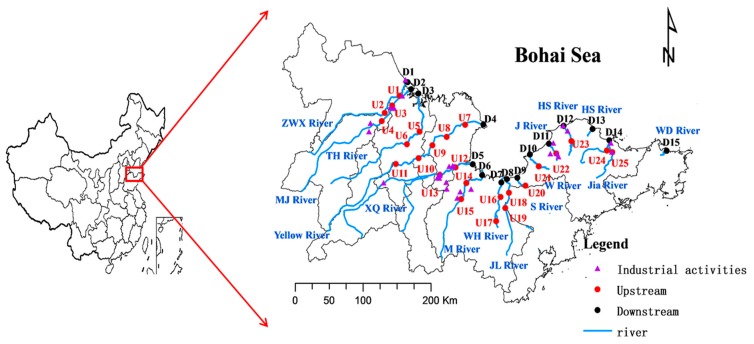
Sampling sites along the watershed of the Bohai Sea (upstream sampling sites are labeled U1 to U25, and downstream sites are labeled D1 to D15).

Study sites were selected along the watershed of the Bohai Sea, between 36.58° to 38.25° N and 117.42° to 122.26° E. Mean annual precipitation in the study area is 740 mm, mostly occurring from July to September, and mean annual air temperature is 13.6 °C. Forty surface sediment samples were collected from 14 main rivers along the southern Bohai Sea during September 2011 (Figure 1). Sediments samples were collected from 15 downstream sites and 25 upstream sites. Throughout the survey, a global positioning system (GPS) was used to locate and map all of the sampling sites. At each sampling point, approximately the top 5 cm of sediments were collected using a sediment sampler. All samples were sealed in clean polyethylene bags and put in a cooled box on site. The cooled samples were brought back to the lab to be freeze-dried, and each sediment sample was divided into two parts. One part was crushed in an agate mortar to pass through a 2.0-mm mesh nylon sieve for the determination of pH and granulometry, and another part was ground to pass through a nylon 100-mesh sieve for the determination of metals and total organic carbon (TOC).

### 2.2. Sample Analysis

Sediments were digested with a mixture of concentrated HCl-HNO_3_-HF-HClO_4_ [16]. Concentrations of the metals Cd, Cu, Ni, Pb, Cr and Zn in the digestion solution were determined by ICP-MS. The detection limits were 0.02 mg/kg for Cd, 0.10 mg/kg for Cr and Cu and 0.50 mg/kg for Ni, Pb and Zn. Standard reference materials (GBW07307), obtained from the Center of National Standard Reference material of China, were analyzed as part of the quality assurance and quality control (QA/QC) procedures. The percentage recoveries for metals ranged from 92% (for Ni) to 108% (for Zn), and the replicate analysis of each batch of samples showed that the analytical precision was within 10% variability. Samples were carefully handled to avoid introduction or loss of trace elements during preparation and analysis. All materials used during analytical determinations were kept in Teflon or other metal-free containers. Sediment TOC was determined by titration with FeSO_4_ after digestion with a K_2_Cr_2_O_7_–H_2_SO_4_ solution. Soil pH was determined from a 1:2.5 water-soil slurry using a pH meter. The sample granulometry was analyzed using a Malvern Mastersizer 2000 laser diffractometer capable of analyzing particle sizes between 0.02 and 20000 um. The percentages of the following 3 groups of grain sizes were determined: <4 µm (clay), 4–63 µm (silt) and >63 µm (sand).

The metals in all of the sediments were fractionated according to the BCR sequential extraction as briefly described below [17]:

Step 1: Weak acid soluble fraction. Each 0.50-g sediment sample was placed in a 50.0-mL polypropylene centrifuge tube containing 20.0 mL of HOAc (0.11 mol·L^−1^) and then shaken for 16 h at room temperature. The solution and solid phase were separated by centrifugation at 3000 rpm for 20.0 min. Subsequently, the suspension was filtered through a 0.45-mm membrane filter. The supernatant was collected for metal measurement, and the solid residues were subjected to the extractions described in Steps 2 to 4.
Step 2: Reducible fraction. Twenty milliliters of 0.50 mol/L NH_2_OH·HCl (with pH of 1.50 adjusted by HNO_3_) were added to the solid residues from Step 1. The mixture was shaken for 16 h at room temperature and then centrifuged and filtered. The supernatant was collected, and the solid residues were subjected to the extractions described in Steps 3 and 4.
Step 3: Oxidizable fraction. Ten milliliters of 30% H_2_O_2_ with a pH value of 2 to 3 (adjusted using HOAc) were added to the residues from Step 2 and digested at 85 °C (water bath) for 1 h with occasional shaking. A second 10.0-mL aliquot of H_2_O_2_ was added to the mixture and digested at 85 °C (water bath) for 1 h with occasional shaking. The mixture was then evaporated to a small volume (1 to 2 mL). Twenty-five milliliters of NH_4_Ac (1.00 mol·L^−1^ and pH = 2 adjusted with HNO_3_) were added to the cooled moist residue and then shaken, centrifuged and filtered. The supernatant was subjected to metal analysis, and the solid residues were used in Step 4.
Step 4: Residual fraction. The same method for analyzing the total concentrations of the metals in soil samples was adopted.

The concentrations of the metals in different fractions and the resultant solutions of Step 4 were determined by ICP-MS. The standard reference material (BCR 701) was used to verify the accuracy of the sequential extraction method. The recovery rates for metals ranged from 92% (for Zn in the exchangeable phase) to 112% (for Cr in the residual fraction).

### 2.3. Statistical Analyses

In this study, frequency distributions of concentrations of trace metals were investigated by calculating coefficients of skewness and kurtosis. A correlation analysis was conducted to identify the correlation coefficients among elements in sediment samples along the river catchments of the southern Bohai Sea. A cluster analysis was performed using Ward’s method based on Euclidean distance. ESRI ArcGIS 10.0 for Windows was used for the area digitization in order to display the spatial distribution of sampling sites along the watershed of the southern Bohai Sea. All mathematical and statistical computations were made using Microsoft Excel and SPSS 16.0 for Windows.

## 3. Results and Discussion

### 3.1. Total Concentration of Metals in Sediments

The concentration range, median, mean, standard deviation (S.D), skew and kurtosis values of Cu, Zn, Cr, Ni, Cd and Pb in the sediments from the upstream and downstream reaches of the river catchments of the southern Bohai Sea are listed in Table 1. For comparison, the average upper continental crust (UCC) values were also shown. Of the six studied metals, as with the average concentrations in the upper continental crust, the mean concentration of Zn was the highest and Cd the lowest. The mean concentration of each metal from the upstream reaches was higher than the corresponding upper continental crust (UCC) value. However, the mean concentrations of Zn and Ni from the downstream reaches were lower than those in the upper continental crust. The standard deviation, skew and kurtosis values for each metal from the upstream reaches were higher than from the downstream reaches, indicating that the variation of each metal in the freshwater system was bigger than in the marine water system. This observation is the result of the heterogeneity of metal contents in sediment from the upstream reach where a few locations have elevated concentrations.

In upstream sediment samples, the TOC content ranged from 0.15% to 3.10% with a mean of 1.29%; in the downstream sediment sample, the TOC content ranged from 0.23% to 1.38% with a mean of 0.77%. The mean upstream and downstream pH values were 7.77 and 7.84, respectively. The average percentages of clay, silt and sand in sediment samples from upstream reaches were 28.7%, 66.3% and 4.96%, respectively; for sediment sites from downstream reaches, the average percentages of clay, silt and sand were 25.5%, 67.6% and 6.92%, respectively. The mean and median concentrations of each metal (except for the median concentration of Pb) from the upstream reaches were higher than in the downstream reaches, indicating higher overall levels of metal contamination in the upstream reaches. In general, metal contamination in the estuary was higher than in the upstream reaches, because slow water flow allowed deposition of particulate matter and fine sediment that can absorb metals. However, the transportation capacity of the rivers along the catchments of the southern Bohai Sea has been decreased by the construction of small dams, leading to low water volume. Secondly, the upstream TOC content was higher than downstream, and sediment that is rich in organic matter can offer more functional groups (such as COO-groups) that can complex and deposit metals ions [18]. Hence, the more organic matter present in sediment, the more metals can be fixed. Thirdly, the sand fraction of sediment from the downstream reach was higher than that from the upstream reach. The increase of sand fraction downstream could have the effect of reducing the concentration of two possible factors: (i) the fine fraction, which usually absorbs the metals, is trapped in dams along river courses; and (ii) the fine sediments were lost offshore, because intermittent hydrological events with high flows may result in erosion/resuspension of the fine fraction. Furthermore, due to soil erosion and the exploitation of the coastline, uncontaminated soil has been brought into the estuary, where it has mixed with polluted sediment, decreasing the concentration of metals in the sediment.

**Table 1:** Metal concentrations in sediments from the upstream and downstream reaches.

Area		Cu	Zn	Cr	Ni	Cd	Pb	pH	TOC	Clay (%)	Silt (%)	Sand (%)
Upstream	Max	1.46 × 10^3^	1.60 × 10^3^	196	67.2	1.41	63.5	9.16	3.10	45.3	74.1	32.5
	Min	1.27	8.10	0.45	1.92	0.07	17.9	6.49	0.15	8.70	54.0	0.69
	Median	20.8	57.9	47.4	23.3	0.14	22.4	7.80	0.98	29.3	66.5	2.36
	Mean	82.6	157	63.6	26.6	0.18	24.9	7.77	1.29	28.7	66.3	4.96
	SD	289	321	484	131	0.26	8.79	0.58	0.81	8.77	5.47	6.91
	Skew	4.94	4.15	1.11	1.02	4.84	3.80	0.16	0.80	−0.37	−0.61	3.08
	Kurtosis	24.6	18.6	0.94	2.96	23.9	16.7	0.59	−0.24	0.26	−0.40	10.6
Downstream	Max	345	361	100	37.2	0.68	36.7	6.96	1.38	37.6	74.1	20.1
	Min	0.22	11.8	1.38	3.36	0.06	16.8	8.56	0.23	13.8	60.7	0.96
	Median	16.9	44.0	38.2	16.2	0.14	22.6	7.83	0.76	25.9	68.0	4.84
	Mean	38.0	66.0	38.9	18.1	0.16	24.0	7.84	0.77	25.5	67.6	6.92
	SD	86.1	83.1	26.4	10.1	0.15	5.65	0.42	0.29	6.70	3.82	6.28
	Skew	3.71	3.63	0.67	0.29	3.55	1.25	−0.32	0.19	0.01	−0.17	1.17
	Kurtosis	14.1	13.7	0.68	−0.79	13.3	1.30	−0.10	0.32	−0.28	−0.64	0.13
UCC ^a^		25.0	71.0	35.0	20.0	0.10	20.0					

^a^ Average concentrations of the Upper Continental Crust [19].

### 3.2. Speciation of Metals

**Figure 2:**
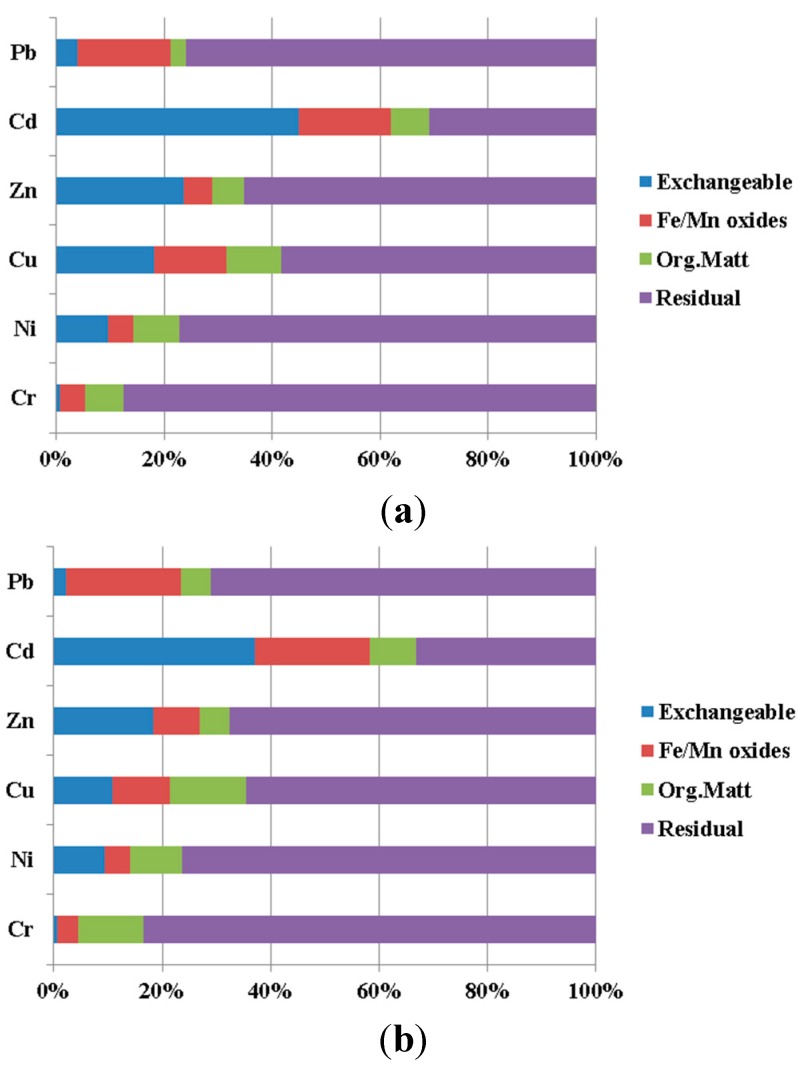
Partitioning of metals in the sediments. (**a**) Downstream; (**b**) upstream.

The metals’ speciation in sediments is important to understand, because it affects metal mobility and availability [8]. In this study, the metals in the upstream and downstream sediments were extracted into four fractions by using the BCR procedure. In the majority of cases, the sums of the extracted fractions agree, to within 15%, with the independently-determined total metal concentrations discussed above, which supports the overall accuracy of the extraction procedure. The percentages of metals in the sediment sample that were extracted in each step of the sequential extraction procedure used in this study are plotted in Figure 2.

As Figure 2 shows, Cr, Ni, Pb, Zn and Cu were preferentially bound to the residual phase. Of the investigated elements, Cr was the most abundant in the residual phase (88.0% and 83.0% for downstream and upstream samples, respectively). The residual fraction is also identified as the stable fraction, which is not expected to be released in solution over an environmentally-relevant time scale [20]. We conclude that Cr is a negligible pollutant in both the upstream and downstream reaches and is mainly derived from natural processes, such as weathering and soil formation [21,22]. Among the investigated elements, Cd was the most abundant in the exchangeable phase. The operationally-defined carrier phases of Cd were as follows: exchangeable > residual phase > Fe/Mn oxide > organic/sulfides. Low levels of Cd in the organic matter/sulfides can be due to the low adsorption constant and labile complex with organic matter [23]. Cadmium has always been considered to be an environmentally-important metal. It can be concluded that potential Cd pollution in the study area should have more attention paid to it.

A comparison of the speciation distribution of metals in upstream and downstream sediments shows that the proportions of the exchangeable phases of Cd, Zn and Cu were higher in the downstream reaches. The pH value plays an important role to control metals’ sorption and desorption [24]. The pH value from the upstream reach was similar to that of the downstream reach. However, the exchangeable phases of Cd, Zn and Cu from downstream reaches were significantly higher than those from upstream reaches. The sediment TOC contents from upstream reaches were higher than those from downstream reaches; however, the percentage of organic/sulfides fraction of metals from upstream reaches were not higher those from downstream reaches. Normally, high TOC content in sediment will lead to a high percentage of organic/sulfides fraction of metals in sediment [25]. Therefore, there must be some factors that changed the distribution of the speciation of metals in the study area. It is well known that the concentrations of salt solutions in the downstream area are higher than those in the upstream area [26]. The elevation in concentration of salt solutions affects the release of sediment-associated metals, especially for metals that have a high affinity for the carbonate fraction [27]. The salt anions, especially Cl^−^, can unavoidably complex and compete with Cd ions to form stable compounds, such as CdCl^+^ and ${\text{CdCl}}_{0}^{2}$ [28]. The stability and solubility of these complexes are higher than the affinity of Cd for the solid soil phase, thus leading to the higher mobility of Cd in the downstream reaches [29]. The exchangeable phases of Cr, Ni and Pb from the downstream sites were similar to those from upstream sites. In every phase, the differences in Ni between upstream and downstream sites were the smallest of all the studied metals. The results indicate that salt solutions have less influence on determining the partitioning of these metals in sediments [30]. Fonseca, *et al.* [31] found that, Pb cations were retained at sites with high sorption energies and that the Pb cations seemed to be sorbed more strongly to sites with high dissociation constants, making them less vulnerable to leaching.

### 3.3. Metal Enrichment and Potential Risk

Sediment quality guidelines (SQGs) are very important for the protection of benthic organisms in aquatic ecosystems and can be used to identify “contaminants of concern” in these ecosystems and to rank “areas of concern” on a regional or national basis [32,33], and they have also been used in numerous other applications [34]. Two sets of sediment quality guidelines were used, one for freshwater sediment and another for marine sediment. For each set, two level of sediment quality guidelines were considered, the threshold effect level (TEL) and the probable effects level (PEL). These two assessment levels were used to delineate three ranges of chemical concentrations that were rarely (minimal effect range; concentrations equal to and below the TEL), occasionally (possible effect range; concentrations above the TEL, but below the PEL) and frequently (probable effect range; concentrations equal to and above the PEL) associated with adverse biological effects [35].

The metal concentrations from upstream and downstream reaches were compared with the corresponding TEL and PEL values, and the results are presented in Table 2. The percentages of upstream samples exceeding the TEL and PEL values for Cr were 44.0% and 24.0%, respectively. The percentages of downstream samples exceeding the TEL and PEL values for Cr were 26.67% and 0%, respectively. Among the investigated elements, Ni most often exceeded the TEL values for both the upstream and downstream reaches. For Pb and Cd, 4.00% of sediment sites from upstream reaches would be expected to occasionally be associated with adverse biological effects on aquatic organisms; for sediments sites from downstream reaches, 13.3% and 6.67% would be expected to occasionally be associated with toxic effects on aquatic organisms due to Pb and Cd, respectively. For Cu, 16.0% and 4.00% of upstream sediment sites would be expected to have adverse biological effects on benthic organisms occasionally and frequently, respectively; for downstream sites, 20.0% and 6.67% would be expected to have adverse biological effects on benthic organisms occasionally and frequently, respectively. The results indicate that 80.0% of upstream sites were below the TEL guideline for Zn, while 8.00% and 12.0% of sites exceeded the TEL and PEL values, respectively. The results also indicate that 93.3% of downstream sites were below the TEL guideline for Zn, and the concentrations of Zn in the remainder of the sites would be classified as frequently presenting an adverse threat to organisms.

**Table 2:** Classification of sediment samples based on the proposed sediment quality guidelines (SQGs). TEL, threshold effect level; PEL, probable effects level.

Metal	SQGs (mg/kg)-Freshwater Sediment (Upstream)		Percentage of Samples Exceeding SQGs (%)		
	TEL	PEL	<TEL	TEL-PEL	>PEL
Cu	35.7	197	80.0	16.0	4.00
Zn	123	315	80.0	8.00	12.0
Cr	37.3	90.0	32.0	44.0	24.0
Ni	18.0	35.9	16.0	68.0	16.0
Pb	35.0	91.3	96.0	4.0	0.00
Cd	0.60	3.53	96.0	4.00	0.00
**Metal**	**SQGs (mg/kg)-Marine Sediment (Downstream)**		**Percentage of Samples Exceeding SQGs (%)**		
	**TEL**	**PEL**	**<TEL**	**TEL-PEL**	**>PEL**
Cu	18.7	108	73.3	20.0	6.67
Zn	124	271	93.3	0.00	6.67
Cr	52.3	160	73.3	26.7	0.00
Ni	15.9	42.8	46.7	53.3	0.00
Pb	30.2	112	86.7	13.3	0.00
Cd	0.68	4.21	93.3	6.67	0.00

### 3.4. Cluster and Correlation Analyses

**Figure 3:**
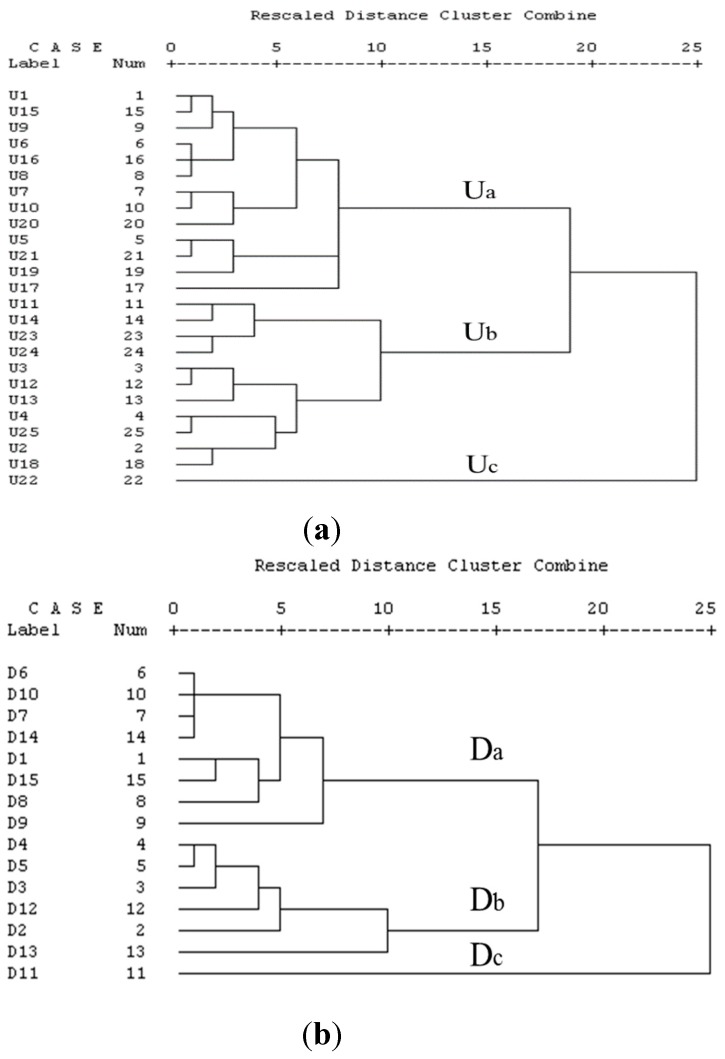
Dendrogram of the cluster analysis of metal concentrations in the study area. (**a**) Upstream (U); (**b**) downstream (D).

A cluster analysis was used to elucidate the latent relationships between metals in sediments along the river catchment of the southern Bohai Sea. The distance cluster represents the degree of association between sites. The smaller value of the distance cluster, the more significant the association. A criterion was adopted for distance clusters requiring that they be between 10 and 15. In sediments of river catchments along the southern Bohai Sea, three distinct clusters were identified in both the upstream and downstream reaches (Figure 3).

Uc (U22) and Dc (D11) represent highly-contaminated sites that have high amounts of Cu, Zn and Cd. Both Uc and Dc are from the same river (Jie River) where eco-toxicological damage might be occurring. This river is located in the gold-mining area where the output of gold was the first of all in China. It is well known that a significant increase in environmental metal concentrations can happen during gold mining, smelting processes and subsequent leaching of tailings [36,37]. Accordingly, this river should be given high priority in initial remediation efforts. The second cluster represent sites of moderate (Ub, Db) heavy metal concentrations in the sediments. This cluster contains the sites affected by suspected major pollution sources, such as chemical plant, harbor and industrial wastewater. The third clusters represented sites of lower (Ua, Da) heavy metal levels in the sediments.

A correlation analysis was conducted to determine the extent of association among metals from upstream and downstream reaches along the catchments of the southern Bohai Sea. The results from the analysis (Table 3) show that combined pollution exists in the sediment. There are significant correlations among Group a (Cu, Cd, Zn and Pb, *r* > 0.90) and Group b (Cr and Zn, *r* = 0.51; Cr and Pb, *r* = 0.53) in the upstream reaches (these metals are commonly associated with industrial and residential activities). There are significant correlations among Cu, Cd and Zn in sediments from downstream reaches. Concentrations of Pb were significantly correlated (*p* < 0.01) with concentrations of Zn and Ni and less correlated (*p* < 0.05) with concentrations of Cu in the downstream reaches. No correlations were noted between Ni and other metals in the two areas, suggesting that Ni contamination might be from a different source than the other metals or that it might have different sediment deposition characteristics.

**Table 3:** Correlation analysis of metals among all sampling sites.

Area		Cu	Zn	Cr	Ni	Cd	Pb	TOC	pH	Clay (%)	Silt (%)	Sand (%)
Upstream	Cu	1.00										
	Zn	0.96 **	1.00									
	Cr	0.34	0.51 **	1.00								
	Ni	0.23	0.25	0.19	1.00							
	Cd	0.99 **	0.94 **	0.35	0.19	1.00						
	Pb	0.94 **	0.92 **	0.53 **	0.23	0.94 **	1.00					
	TOC	0.47 *	0.52 *	0.59 *	0.35	0.48 *	0.55 *	1.00				
	pH	−0.49 *	−0.57 **	−0.46 *	−0.29	−0.48 *	−0.50 *	−0.55 **	1.00			
	Clay (%)	0.45 *	0.56 **	0.77 **	0.22	0.47 *	0.53 **	0.62 **	−0.51 **	1.00		
	Silt (%)	−0.51 **	−0.61 **	−0.67 **	−0.22	−0.50 *	−0.60 **	−0.58 **	0.69 **	−0.62 **	1.00	
	Sand (%)	−0.16	−0.22	−0.45 *	−0.11	−0.20	−0.20	−0.33	0.10	−0.78 **	−0.01	1.00
**Area**		**Cu**	**Zn**	**Cr**	**Ni**	**Cd**	**Pb**	**TOC**	**pH**	**Clay (%)**	**Silt (%)**	**Sand (%)**
Downstream	Cu	1.00										
	Zn	0.98 **	1.00									
	Cr	0.11	0.15	1.00								
	Ni	−0.01	0.10	0.48	1.00							
	Cd	0.97 **	0.99 **	0.16	0.03	1.00						
	Pb	0.61 *	0.66 **	−0.10	0.23	0.65 **	1.00					
	TOC	0.14	0.25	0.11	0.56 *	0.20	0.30	1.00				
	pH	−0.59 *	−0.54 *	−0.08	0.37	−0.54	−0.15	0.00	1.00			
	Clay (%)	−0.06	0.10	0.46	0.35	0.03	0.80	−0.07	−0.17	1.00		
	Silt (%)	0.24	0.24	−0.09	−0.26	0.29	−0.06	−0.13	−0.20	−0.40	1.00	
	Sand (%)	−0.08	−0.16	−0.44	−0.21	−0.20	−0.05	0.15	0.30	−0.83 **	−0.20	1.00

** Correlation is significant at the 0.01 level (2-tailed); * correlation is significant at the 0.05 level (2-tailed).

The relationships between TOC, pH and metals are also shown in the Table 3. TOC was less correlated (*p* < 0.05) with any of the metals, except Ni, in the upstream reaches. The pH values were significantly negatively correlated (*p* < 0.01) with Zn and less negatively correlated (*p* < 0.05) with Cu, Cd, Cr and Pb in the upstream reaches. In the downstream reaches, TOC was not strongly correlated (*p* < 0.05) with Ni; pH was negatively correlated (*p* < 0.05) with Cu and Zn. In addition, much of the literature showed that metal accumulation and distribution in sediments follows grain size patterns in a natural environment [38,39,40]. Therefore, the relationships between grain size fraction and metal concentration are also discussed (Table 3). The clay content was less correlated (*p* < 0.05) with Cu and Cd and significantly correlated (*p* < 0.01) with Zn, Cr and Pb in the upstream reaches. The silt content was significantly negatively correlated (*p* < 0.01) with Cu, Zn, Cr and Pb and less negatively correlated (*p* < 0.05) with Cd in the upstream reaches. The sand content was negatively correlated (*p* < 0.05) with Cr in the upstream reaches. In the downstream reaches, silt and sand content were not correlated with any of the metals. Based on these results, we conclude that TOC, pH, clay and silt content play more important roles in determining the concentrations of metals in the upstream than in the downstream ones, which also indicates that other factors in the downstream sites, such as salinity, might have a stronger affinity effect on metals than do TOC, pH and clay content.

## 4. Conclusions

Speciation and degrees of contamination of metals in upstream and downstream sediments along the southern Bohai Sea were studied. The mean and median concentrations of Cu, Zn, Cr, Ni and Cd from the upstream reaches were higher than those in the downstream reaches. Cu, Zn, Ni, Pb and Cr were mainly bound to the residual phase; however, Cd was the most abundant in the exchangeable phase. The proportions of the exchangeable phase of Cu, Zn and Cd from the downstream reaches were higher than those in the upstream reaches. Highly contaminated sites (U22 and D11) were identified by cluster analysis; both of these two sites were from the same river (Jie River) where they were affected by gold mining, smelting processes and subsequent leaching of tailings. Correlation analysis indicated that TOC, pH and clay content play more important roles in determining the concentrations of metals in the upstream than in the downstream ones. According to sediment quality guidelines, although concentrations of Cu, Pb and Cd were lower in downstream reaches than in upstream reaches, those three metals in sediments from downstream reaches present a higher risk than in upstream reaches.
